# A modified filter nonmonotone adaptive retrospective trust region method

**DOI:** 10.1371/journal.pone.0253016

**Published:** 2021-06-17

**Authors:** Xianfeng Ding, Quan Qu, Xinyi Wang

**Affiliations:** 1 School of Sciences, Southwest Petroleum University, Chengdu, China; 2 School of Artificial Intelligence, Southwest Petroleum University, Chengdu, China; University of Zaragoza, SPAIN

## Abstract

In this paper, aiming at the unconstrained optimization problem, a new nonmonotone adaptive retrospective trust region line search method is presented, which takes advantages of multidimensional filter technique to increase the acceptance probability of the trial step. The new nonmonotone trust region ratio is presented, which based on the convex combination of nonmonotone trust region ratio and retrospective ratio. The global convergence and the superlinear convergence of the algorithm are shown in the right circumstances. Comparative numerical experiments show the better effective and robustness.

## 1. Introduction

Consider the following unconstrained optimization problem

$$\underset{x\in R^{n}}{\min}f(x)$$ (1)

where *f*:*R*^*n*^→*R* is a twice continuously differentiable function.

Trust region method and line search method are two effective methods to solve unconstrained optimization problems. At present, the both numerical methods mentioned above for solving nonlinear programming are widely used in many applications of various engineering design, automation, transportation, economic analysis, pattern recognition, artificial intelligence, network design and many other modern high-tech research and development.

Compared with the line search method, the trust region method has novel idea, strong convergence and stable numerical performance, see [1–4]. It can not only solve the well-conditioned problem quickly, but also solve the ill-conditioned optimization problem effectively. The basic idea of trust region method as follows: at the iteration point *x*_*k*_, the trial step *d*_*k*_ is obtained by solving the subproblem.

$$\underset{d\in R^{n}}{\min}m_{k}(d)=g_{k}^{T}d+\frac{1}{2}d^{T}B_{k}d$$ (2)

$$\|d_{k}\|\leq \Delta_{k}$$

*g*_*k*_ denotes by ∇_*f*_(*x*_*k*_), *B*_*k*_ is a symmetric approximation of ∇^2^*f*(*x*_*k*_), Δ_*k*_ stands for the trust region radius, and ||.|| shows any vector norm, usually the Euclidean norm.

To evaluate the consistency between the quadratic model and the objective function, the most classical ratio, denoted by $\rho_{k}^{B}$, is defined as follows,

$$\rho_{k}^{B}=\frac{f(x_{k})-f(x_{k}+d_{k})}{m_{k}(0)-m_{k}(d_{k})}$$ (3)

The trial step *d*_*k*_ is accepted whenever $\rho_{k}^{B}$ is closed to 1, that is to say *x*_*k*+1_ = *x*_*k*_+*d*_*k*_, and Δ_*k*_ is updated suitably. Otherwise, $\rho_{k}^{B}$ is negative or positive but not close to 1, Δ_*k*_ is decreased and the subproblem should be resolved.

It is well-known that monotone techniques may not only lead to the rate of convergence slows down, especially in the presence of the narrow curved valley, but also the objective function is required to be decreased at each iteration. Considering this fact, it is most meaningful to study nonmonotone technique for improving algorithm. Obviously, the first nonmonotone technique was the alleged watchdog technique proposed by Chamberlain et al. [5], which was designed to overcome the Maratos effect. At the same time, nonmonotone techniques in [6–8] have attracted extensive attention from scholars, for example, Deng et al. [9] proposed a nonmonotone trust region method by replacing *f*(*x*_*k*_) in (3) with *f*_*l*(*k*)_ given by

$$f_{l(k)}=f(x_{l(k)})=\underset{0\leq j\leq m(k)}{\max}\{f_{k-j}\}$$ (4)

where *f*_*i*_ = *f*(*x*_*i*_), *m*(0) = 0, 0≤*m*(*k*)≤min{*N*, *m*(*k*−1)+1}, and *N*≥0 is an integer constant. Deng et al. [9] proposed another nonmonotone trust region method with the following ratio.


$${\overset{⌢}{\rho }}_{k}=\frac{f_{l(k)}-f(x_{k}+d_{k})}{m_{k}(0)-m_{k}(d_{k})}$$ (5)

Motivated by this, Ahookhoosh et al. [6] proposed a nonmonotone trust region method with the following ratio,

$${\overset{⌣}{\rho }}_{k}=\frac{R_{k}-f(x_{k}+d_{k})}{m_{k}(0)-m_{k}(d_{k})}$$ (6)

where

$$R_{k}=\eta_{k}f_{l(k)}+(1-\eta_{k})f_{k}$$ (7)

in which *η*_*k*_∈[*η*_min_,*η*_max_] with *η*_min_∈[0,1) and *η*_max_∈[*η*_min_,1].

After that, Grippo et al. [10] proposed a nonmonotone line search technique for Newton’s method, that is,

$$f(x_{k}+\alpha_{k}d_{k})\leq f_{l(}{}_{k)}+\sigma \alpha_{k}g_{k}^{T}d_{k}$$

However, Grippo’s nonmonotone technique has a drawback in that numerical performance is highly dependent on the choice of *N*. It makes more sense, for given *σ*∈(0,1), the step size *α*_*k*_ is chosen so that

$$f(x_{k}+\alpha_{k}d_{k})\leq R_{k}+\sigma \alpha_{k}g_{k}^{T}d_{k}$$ (8)

As is well-known, an appropriate updating strategy of trust region radius plays a valuable role in a manner that it may prominently affect the computational efficiency. Motivated by this, the versions of trust region radius have attracted considerable attention from many scholars in [11–13]. In order to avoid the gradient or the Hessian information is not precisely employed in the standard trust region, Zhang et al. [14] proposed a new scheme, which use the following trust region radius,

$$\Delta_{k}=c^{p}\|g_{k}\|\|{\bar{B}}_{k}^{-1}\|$$ (9)

where *c* is a constant, *p* is an adjustment parameter, ${\bar{B}}_{k}=B_{k}+iI$, *i*∈*N*.

Despite effectiveness of the Zhang’s method, calculating an estimation of the inverse of the Hessian in each iteration which results in some additional computational costs. Qu et al. [15] refered to another adaptive strategy for updating the trust region radius as follows,

$$\Delta_{k}=c^{p}{\left\|g_{k}\right\|}^{\gamma }$$ (10)

Here, the filter technique introduced by Fletcher and Leyffer [16], which avoids the difficulty of updating the penalty parameter in penalty functions. The filter is able to reject poor trial iterates and enforce global convergence from arbitrary starting points. In this case, it is worth mentioning that Gould et al. [17] proposed an algorithm by using filter technique for unconstrained optimization problems, the main idea is to accept the new iteration point as much as possible.

The filter is primarily composed of a series of gradient of iteration points, the so-called trial step is accepted by the filter, in fact, the corresponding gradient is accepted by the filter. Set ∇*f*(*x*_*k*_) = *g*(*x*_*k*_) = *g*_*k*_ = (*g*_*k*,1_,*g*_*k*,2_,…,*g*_*k*,*n*_), *g*_*k*,*i*_ (*i* = 1,2,…,*n*) is the i-th component of *g*_*k*_. We say that an iterate *x*_1_ dominates *x*_2_, whenever

$$|g_{1,i}|\leq |g_{2,i}|\;\forall \;i=1,2,\ldots ,n$$

The basic concept of multidimensional filter F is a list of *n*–tuples of the form (*g*_*k*,1_,*g*_*k*,2_,…,*g*_*k*,*n*_). Suppose that $g_{k}\in F$, $g_{l}\in F$, there exists *j*∈(1,2,…*n*) such that

$$\left|g_{k,j}|\leq |g_{l,j}\right|$$

Subsequently, compared with [17], in order to maximize the possibility of acceptance of the trial point, we introduced an improved multidimensional filter F, specifically as follows,

Set $\phi_{l}=\min\{\left\|g_{l}\right\||(g_{l,1},g_{l,2},\ldots ,g_{l,n})\in F\}$, a new trial point *x*_*k*_ is acceptable if there exists *j*∈{1,2,3,…*n*}, such that

$$|g_{k,j}|\leq \underset{1\leq j\leq n}{\max}\|g_{l,j}\|-\gamma_{g}\phi_{l},$$ (11)

When an iteration point *x*_*k*_ is accepted, we add *g*_*k*_ to the filter and Meanwhile and remove the points which are dominated *x*_*k*_ by from the filter.

The rest of the paper is organized as follows. Section 2 is devoted to describe the new filter nonmonotone adaptive restrospective trust region method in details. The global convergence and superlinear convergence are established in Section 3. Some preliminary numerical results are introduced in Section 4. Finally, some conclusions are summarized in Section 5.

## 2. Materials and methods

In this section, we propose a new filter nonmonotone adaptive retrospective trust region algorithm for solving unconstrained optimization problems. In order to reduce the computational cost, at the iteration point *x*_*k*_, the new nonmonotone ratio and retrospective ratios are introduced based on [13] as follows:

$$\rho_{k}^{NB}=\frac{R_{k}-f(x_{k}+d_{k})}{\varepsilon_{k}f_{l(k)}+m_{k}(0)-m_{k}(d_{k})}$$ (12)

$$\rho_{k+1}^{NR}=\frac{R_{k}-f(x_{k}+d_{k})}{\varepsilon_{k+1}f_{l(k+1)}+m_{k+1}(0)-m_{k+1}(d_{k})}$$ (13)

where *ε*_*k*_∈[0,*η*_*k*_]. As can be seen from the motivation of this nonmonotone term *R*_*k*_, the better convergence result can be obtained by freely selecting the parameter *η*_*k*_ and *ε*_*k*_. As a result of above discussion, a new nonmonotone ratio is introduced to improve computational efficiency through the convex combination of $\rho_{k}^{NB}$ and $\rho_{k+1}^{NR}$, i,e.

$$\rho_{k+1}^{NC}=\gamma \rho_{k}^{NB}+(1-\gamma )\rho_{k+1}^{NR}$$ (14)

where *γ*∈[*γ*_min_,*γ*_max_]⊂[0,1]. More exactly, $\rho_{k}^{NB}$ is used to determine whether the trial step is acceptable, while $\rho_{k+1}^{NC}$ is employed for updating trust region radius.

In the criteria presented earlier, the Hessian matrix is time-consuming to calculate and the convergence rate of the adaptive trust region method will be less efficient. Inder to reduce the workload and computational time, some simpler information of known iteration points can be used to reconstruct the regulation formula of trust region radius.

In this way, the trust region radius adjustment formula takes into account the gradient information of the function and the solution of the trust region subproblem, which ensures the calculation accuracy of the algorithm is not reduced. The improved adaptive trust region radius as follows,

$$\Delta_{k+1}≔\tau_{k+1}\frac{\left\|g_{k}\|\|d_{k}\right\|}{\left\|g_{k+1}-g_{k}\right\|}$$ (15)

Note that *τ*_*k*_ is computed by the following formula,

$$\tau_{k+1}=\{\begin{array}{ll} \min\{\beta_{2}\tau_{k},\tau_{\max}\} & \mathrm{If}\;\rho_{k+1}^{NC}\geq \mu_{2}\\ \tau_{k} & \mathrm{If}\;\mu_{1}\leq \rho_{k+1}^{NC}<\mu_{2}\\ \beta_{1}\tau_{k} & \mathrm{If}\;\rho_{k+1}^{NC}<\mu_{1} \end{array}$$ (16)

More formally, the new Algorithm is described as follows.

**Algorithm 2.1** (Nonmonotone Adaptive Filter Retrospective Trust Region Method)

**Step 0**. Give *x*_0_∈*R*^*n*^, Δ_max_>0, *B*_0_∈*R*^*n*^×*R*^*n*^, *ε*>0, $\gamma_{g}\in (0,\frac{1}{\sqrt{n}})$, *ϑ*>0, 0<*μ*_1_<*μ*_2_<1, *τ*_0_>0, 0<*β*_1_<1<*β*_2_. Set *F* = ∅, *k*≔0.

**Step 1**. If *k* = 0, set Δ_*k*_ = min{*τ*_*k*_‖*g*_*k*_‖,Δ_max_}, then go to Step 2.

    If $\rho_{k-1}^{NB}<\mu_{1}$, then set *τ*_*k*_ = *β*_1_*τ*_*k*−1_ and $\Delta_{k}=\beta_{1}\tau_{k-1}\frac{\left\|g_{k-1}\|\|d_{k-1}\right\|}{\left\|g_{k}-g_{k-1}\right\|}$, go to Step 2.

    Else, compute $\rho_{k}^{NR}$ and $\rho_{k}^{NC}$ by (13) and (14), respectively. *τ*_*k*_ is updated by (16) and $\Delta_{k}=\min\{\tau_{k}\frac{\left\|g_{k-1}d_{k-1}\right\|}{\left\|g_{k}-g_{k-1}\right\|},\Delta_{\max}\}$.

**Step 2**. Compute ‖*g*_*k*_‖. If ‖*g*_*k*_‖≤*ε*, then stop.

**Step3**. Solve the subproblem (2) to find the trial step *d*_*k*_, set $x_{k}^{+}=x_{k}+d_{k}$.

**Step4**. Compute *R*_*k*_ and $\rho_{k}^{NB}$, respectively.

**Step5**. If $\rho_{k}^{NB}\geq \mu_{1}$, set $x_{k+1}=x_{k}^{+}$. Otherwise, compute $g_{k}^{+}=\nabla f(x_{k}^{+})$, at the same time, if $x_{k}^{+}$ is accepted by the filter F, then $x_{k+1}=x_{k}^{+}$, add $g_{k}^{+}$ into the F, otherwise, find the step size *α*_*k*_ satisfying (8), set *x*_*k*+1_ = *x*_*k*_+*α*_*k*_*d*_*k*_.

**Step6**. Update the symmetric matrix *B*_*k*_ by (30). Set *k* = *k*+1, and go to Step 1.

In this way, it is not necessary to precisely solve the subproblem in Algorithm 2.1, as a result, an approximation of *d*_*k*_ satisfies

$$m_{k}(0)-m_{k}(d_{k})\geq \vartheta \|g_{k}\|\min\{\Delta_{k},\frac{\left\|g_{k}\right\|}{\left\|B_{k}\right\|}\}$$ (17)

$$g_{k}^{T}d_{k}\leq -\vartheta \|g_{k}\|\min\{\Delta_{k},\frac{\left\|g_{k}\right\|}{\left\|B_{k}\right\|}\}$$ (18)

where *ϑ*∈(0, 1)

Assumption 2.1.

1. The level set $L(x_{0})=\{x\in R^{n}|f(x)\leq f(x_{0})\subseteq \Omega \}$ is closed and bounded. *f*(*x*) is a twice continuously differentiable on the level set *L*(*x*_0_), our claim that in a neighborhood *N* of Ω, ∇*f*(*x*) is Lipschitz continuous, there exists a positive constant *L* such that
‖∇f(x)−∇f(y)‖≤L‖x−y‖∀x,y∈Ω
2. The matrix *B*_*k*_ is uniformly bounded, i.e., there exists a constant *M*_1_>0 such that ‖*B*_*k*_‖≤*M*_1_.

## 3. Convergence analysis

In order to ease of operation, the following index sets are defined as follows: $D=\{k|\rho_{k}^{NB}\geq \mu_{1}\}$, $A=\{k|g(x_{k}^{+})\;\mathrm{or}\;x_{k}^{+}\;\mathrm{is}\;\mathrm{added}\;\mathrm{to}\;\mathrm{the}\;\mathrm{filter}\;F\}$, *S* = {*k*|*x*_*k*+1_ = *x*_*k*_+*d*_*k*_}. Then, $S=\{k|\rho_{k}^{NB}\geq \mu_{1}\;\mathrm{or}\;x_{k}^{+}\;\mathrm{is}\;\mathrm{added}\;\mathrm{to}\;\mathrm{the}\;\mathrm{filter}\;F\}$. When *k*∉*S*, we have *x*_*k*+1_ = *x*_*k*_+*α*_*k*_*d*_*k*_.

**Lemma 3.1**. For all *k*, it makes sense for me that

$$\left|f_{k}-f(x_{k}+d_{k})-(m_{k}(0)-m_{k}(d_{k}))|\leq (O{\left\|d_{k}\right\|}^{2}\right)$$ (19)

**Proof**. Motivated by the Taylor’s expansion and Assumption 2.1, we have

$$\begin{array}{l} \;|f_{k}-f(x_{k}+d_{k})-(m_{k}(0)-m_{k}(d_{k}))|\\ =|f_{k}-(f_{k}+g_{k}^{T}d_{k}+\int_{0}^{1}d_{k}^{T}[g(x_{k}+\xi d_{k})-g_{k}]d\xi )+g_{k}^{T}d_{k}+\frac{1}{2}d_{k}^{T}B_{k}d_{k}|\\ \leq |\frac{1}{2}d_{k}^{T}B_{k}d_{k}+\int_{0}^{1}\|d_{k}\|\|g(x_{k}+\xi d_{k})-g_{k}\|d\xi |\\ \leq \frac{1}{2}\|d_{k}{\|}^{2}M_{1}+L\int \|d_{k}\|d\xi \\ =\frac{1}{2}\|d_{k}{\|}^{2}M_{1}+\frac{1}{2}L\|d_{k}{\|}^{2}\\ =O({\left\|d_{k}\right\|}^{2}) \end{array}$$

This completes the proof of the Lemma 3.1.

**Lemma 3.2**. Suppose that Assumption 2.1 holds, the sequence {*x*_*k*_} is generated by Algorithm 2.1. Moreover, assume that there exists a constant 0<*ε*<1, so that ‖*g*_*k*_‖>*ε*, Then, for any *k*, there exists a nonnegative integer *p* such that *x*_*k*+*p*+1_ is a successful iteration point, i.e., $\rho_{k+p}^{NB}\geq \mu_{1}$.

**Proof.** On the contrary, that is, assume that there is an iteration *k* at which *x*_*k*+*p*+1_ is unsuccessful, for an arbitrary nonnegative integer *p*, we have

$$\rho_{k+p}^{NB}<\mu_{1},\;p=0,1,2,\ldots .$$ (20)

This clearly implies that

$$\Delta_{k+p+1}=\beta_{1}^{p+1}\tau_{k}\frac{\left\|g_{k+p}\|\|d_{k+p}\right\|}{\left\|g_{k+p+1}-g_{k+p}\right\|}\leq \beta_{1}^{p+1}\tau_{\max}\frac{\left\|g_{k+p}\|\|d_{k+p}\right\|}{\left\|g_{k+p+1}-g_{k+p}\right\|}$$ (21)

Thus, according to 0<*β*_1_<1 and Eq (21), we have

$$\underset{p\to \infty }{\lim}\Delta_{k+p+1}=0$$ (22)

Now, according to Lemma 3.1, and Eq (17), we get

$$\begin{array}{l} \left|\frac{f(x_{k+p})-f(x_{k+p}+d_{k+p})}{\varepsilon_{k}f_{l(k)}+m_{k+p}(0)-m_{k+p}(d_{k+p})}-1|\leq |\frac{f(x_{k+p})-f(x_{k+p}+d_{k+p})}{m_{k+p}(0)-m_{k+p}(d_{k+p})}-1\right|\\ \;=|\frac{f(x_{k+p})-f(x_{k+p}+d_{k+p})-(m_{k+p}(0)-m_{k+p}(d_{k+p}))}{m_{k+p}(0)-m_{k+p}(d_{k+p})}|\\ \;\leq \frac{O({\left\|d_{k+p}\right\|}^{2})}{\vartheta \|g_{k+p}\|\min\{\Delta_{k+p},\frac{\left\|g_{k+p}\right\|}{\left\|B_{k+p}\right\|}\}}\\ \;\leq \frac{O({\left\|d_{k+p}\right\|}^{2})}{\vartheta \varepsilon \min\{\Delta_{k+p},\frac{\varepsilon }{M_{1}}\}}\\ \;\leq \frac{O(\Delta_{k+p}^{2})}{O(\Delta_{k+p})}\to 0(p\to \infty ) \end{array}$$

Following the definition of *R*_*k*_, we get *R*_*k*_≥*η*_*k*_*f*_*k*_+(1−*η*_*k*_)*f*_*k*_ = *f*_*k*_. Thus, for sufficiently large *p*, we have

$$\frac{R_{k+p}-f(x_{k+p}+d_{k+p})}{\varepsilon_{k}f_{l(k)}+m_{k+p}(0)-m_{k+p}(d_{k+p})}\geq \frac{f(x_{k+p})-f(x_{k+p}+d_{k+p})}{\varepsilon_{k}f_{l(k)}+m_{k+p}(0)-m_{k+p}(d_{k+p})}\geq \mu_{1}$$ (23)

which contradicts (20). This completes the proof of the Lemma 3.3.

**Lemma 3.3.** Suppose that the infinite sequence {*x*_*k*_} is generated by Algorithm 2.1. The number of successful iterations is infinite, that is, |*S*| = +∞. Then, we have {*x*_*k*_}⊂*L*(*x*_0_), meanwhile, the sequence {*f*_*l*(*k*)_} is not monotonically increasing and convergent.

**Proof.** The proof follows the process of lemma 3 and Lemma 4 in [15].

**Lemma 3.4**. Suppose that Assumption 2.1 holds, and there exists a constant *ε* such that ‖*g*_*k*_‖≥*ε* for all *k*. Therefore, there is a constant *υ* such that

$$\Delta_{k}>\upsilon .$$

**Proof.** The proof is similar to the proof of Theorem 6.4.3 in [18].

**Theorem 3.1.** Suppose that Assumption 2.1 holds, and Algorithm 2.1 generates an infinite sequence {*x*_*k*_} which satisfies

$$\underset{k\to \infty }{\lim}\inf\|g_{k}\|=0$$ (24)

**Proof.** The standard of proof can be classified into the following both cases.

Case 1. There is no limit to the number of successful iterations, that is |*S*| = +∞. Meanwhile, there are many infinitely filter iterations, i.e, |*A*| = +∞.

Prove by contradiction, assuming that Eq (24) is not true, then there exists a positive constant *ε* such that ‖*g*_*k*_‖>*ε*. On account of Assumption 2.1, {‖*g*_*k*_‖} is bounded. Set the index in set *A* is the sequence {*k*_*i*_}.Therefore, there exists a subsequence {*k*_*t*_}⊆{*k*_*i*_} which satisfies

$$\underset{t\to \infty }{\lim}g_{k_{t}}=g_{\infty },\;g_{\infty }\geq \varepsilon$$ (25)

This fact, along with the definition of *k*_*t*_, leads to $g(x_{k_{t}})$ is accepted by the filter. Then there exists *j*∈{1,2,…,*n*}, for ∀ *t*>1, that is to say

$$|g_{k_{t},j}|-\underset{1\leq j\leq n}{\max}|g_{k_{t-1},j}|\leq -\gamma_{g}\Phi_{k_{t-1}}$$ (26)

By the fact that $0\leftarrow |g_{k_{t},j}|-\underset{1\leq j\leq n}{\max}|g_{k_{t-1},j}|\leq -\gamma_{g}\varepsilon <0$, as *t* is sufficiently large, which is a contradiction.

Case 2. There is no limit to the number of successful iterations, that is |*S*| = +∞. Meanwhile, there are many finite filter iterations, i.e, |*A*|<+∞.

Contrarily, suppose that the Eq (24) is not true. Then there exists a positive constant 0<*ε*<1 such that ‖*g*_*k*_‖>*ε*, for all *k*.

As a consequence of |*A*|<+∞, for sufficiently large *k*∈*S*, we have $\rho_{k}^{NB}\geq \mu_{1}$. Set

$$\xi_{k}=|\{p,p+1,\ldots ,k\}\cap S|$$

Following the definition of *R*_*k*_, Lemma 3.4 and Eq (17), without loss of generality, we have

$$\sum_{j=p,j\in S}^{k}\left(f_{l(j)}-f_{j+1}\right)\geq \sum_{j=p,j\in S}^{k}\left(R_{j}-f_{j+1}\right)\geq \xi_{k}\vartheta \mu_{1}\varepsilon \min\{\upsilon ,\frac{\varepsilon }{M_{1}}\}\to \infty$$ (27)

Obviously, when *p* is fixed and *k*→∞, $\xi_{k}\vartheta \mu_{1}\varepsilon \min\{\upsilon ,\frac{\varepsilon }{M_{1}}\}\to \infty$ is obtained by *ξ*_*k*_→∞. It’s not surprising that the left end of Eq (27) has no lower bound. Now, we have

$$\begin{array}{l} \sum_{j=p,j=s}^{k}\left(f_{l(j)}-f_{j+1}\right)\geq \sum_{j=p}^{k}\left(f_{l(j)}-f_{l(j+1)}\right)\\ \;=f_{l(p)}-f_{l(k+1)} \end{array}$$ (28)

It indicates that there is a lower limit on the left end of Eq (28), which is a contradiction with Eq (27).

**Theorem 3.2.** Suppose that Assumption 2.1 holds, and {*x*_*k*_} is the sequence generated by Algorithm 2.1 converges to x*. On the other hand, $d_{k}=-B_{k}^{-1}g_{k}$ is a solution of the subproblem, and ∇^2^*f*(x*) is a positive definite matrix. *B*_*k*_ satisfies the following condition

$$\underset{k\to \infty }{\lim}\frac{\left\|(B_{k}-\nabla^{2}f(x^{*})d_{k}\right\|}{\left\|d_{k}\right\|}=0$$ (29)

Then the sequence {*x*_*k*_} converges to x* superlinearly.

**Proof.** The proof follows the same path as given in the proof of Theorem 4.1 in [19].

## 4. Preliminary numerical experiments

In this section, our purpose is to investigate the computational results of algorithm 2.1 on some middle-large size test problems from Andrei [20]. All algorithms are implemented in MATLAB (R2018a) on a PC Intel(R) Core(TM) i7-4558u CPU @2.80GHz 2.80 GHz 4.00 GB RAM memory and double precision format, the following notations represent the different algorithms.

ANTRL: ANTRL method is denoted by Ahookhosh et al. in [6];

NFTR: NFTR method is denoted by [17];

WA FTR: WAFTR method is expressed by Qu et al. in [15];

NAFRTR-1: Algorithm 2.1 with Δ_0_ = 1;

NAFRTR-2: Algorithm 2.1 with Δ_0_ = 10;

NAFRTR-3: Algorithm 2.1 with Δ_0_ = 100;

As is known to all, BFGS correction is one of the most important methods in quasi-Newton method. Several improved BFGS methods are given in [21, 22], and the convergence theory has been well established. More specifically, *B*_*k*+1_ is revised by [23]

$$B_{k+1}=B_{k}-\frac{B_{k}d_{k}d_{k}^{T}B_{k}}{d_{k}^{T}B_{k}d_{k}}+\frac{y_{k}^{m*}y_{k}^{m*T}}{d_{k}^{T}y_{k}^{m*}}$$ (30)

where $y_{k}^{m*}=y_{k}+\frac{\rho_{k}}{{\left\|d_{k}\right\|}^{2}}d_{k}$, and *ρ*_*k*_ = 2(*f*(*x*_*k*_)−*f*(*x*_*k*+1_))+(*g*(*x*_*k*+1_)+*g*(*x*_*k*_))^*T*^*d*_*k*_. It is easy to know that the formula has not only gradient value information, but also function value information. The parameters of these algorithms are exploited identically as follows: *μ*_1_ = 0.25, *μ*_2_ = 0.75, *N* = 5, *β*_1_ = 0.25, *β*_2_ = 1.5, Δ_max_ = 100. It is worth mentioning that the stopping criterion is either ‖*g*_*k*_‖≤10^−6^, or the number of iterations exceeds 10,000. As menthoded in [6], set *η*_0_ = 0.25, the updating principle of *η*_*k*_ follows the following formula,

$$\eta_{k}=\{\begin{array}{ll} \eta_{0}/2, & \mathrm{if}\;k=1\\ (\eta_{k-1}+\eta_{k-2})/2, & \mathrm{if}\;k\geq 2 \end{array}$$ (31)

For the sake of simplicity, we draw efficiency comparisons involved in the number of function evaluations (*n*_*f*_), the number of gradient function evaluations (*n*_*i*_), and the running time (CPU) by using the Dolan-Moré performance profile, in particular, the particulars are detailed in [24]. To account for this, we can choose a performance index as a comparison metric between the above algorithms. For every *τ*≥1, the proportion *ρ*(*τ*) of the test problems is given by the performance profile. The performance of each considered algorithmic variant was the best within a range of *τ* of the best as well.

**Fig 1:**
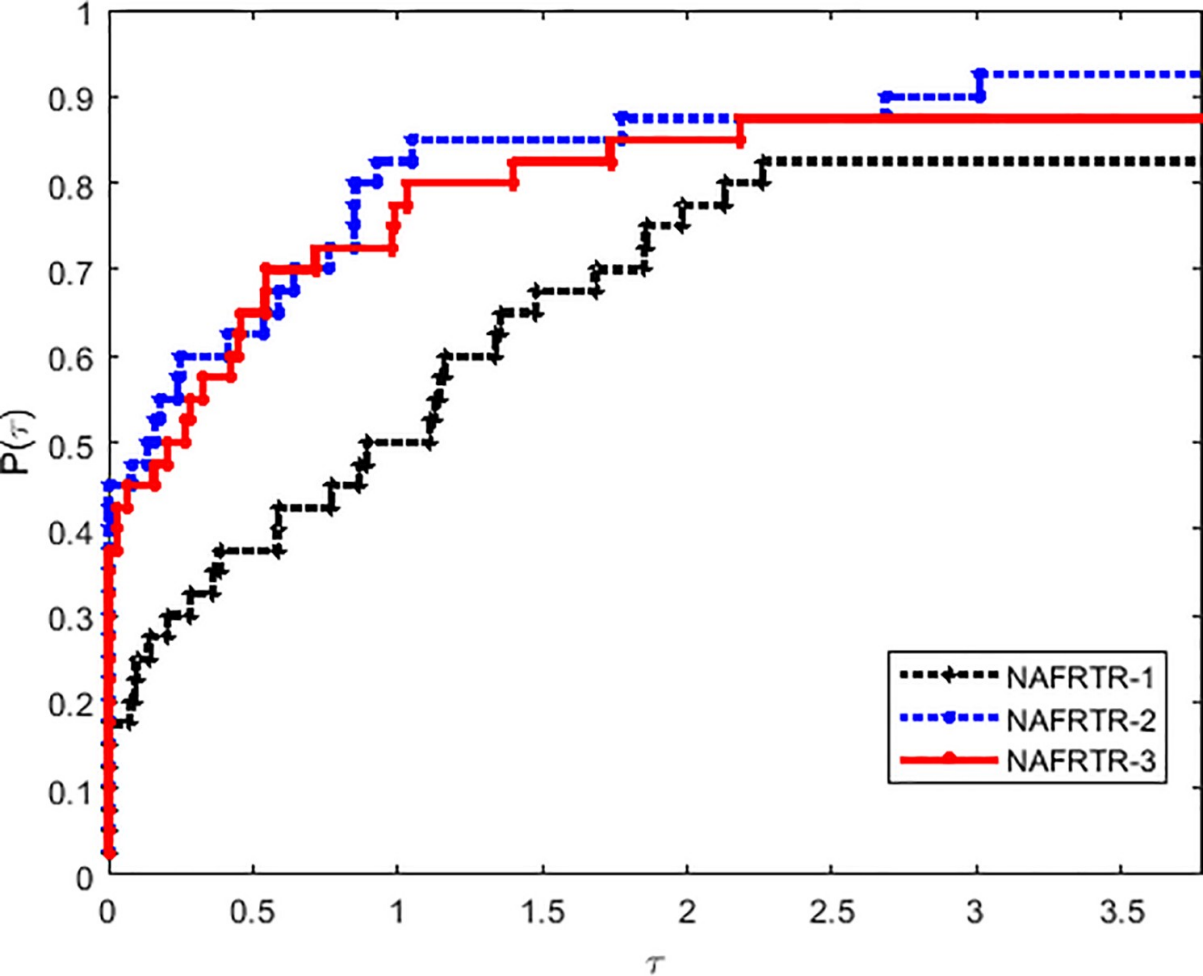
Performance profiles on the basis of running time (in seconds).

**Fig 2:**
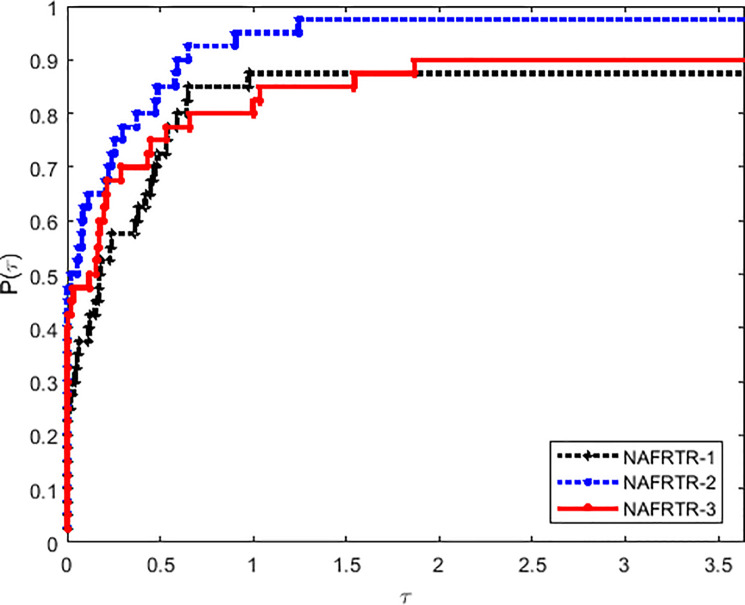
Performance profiles on the basis of the number of function evaluations.

**Fig 3:**
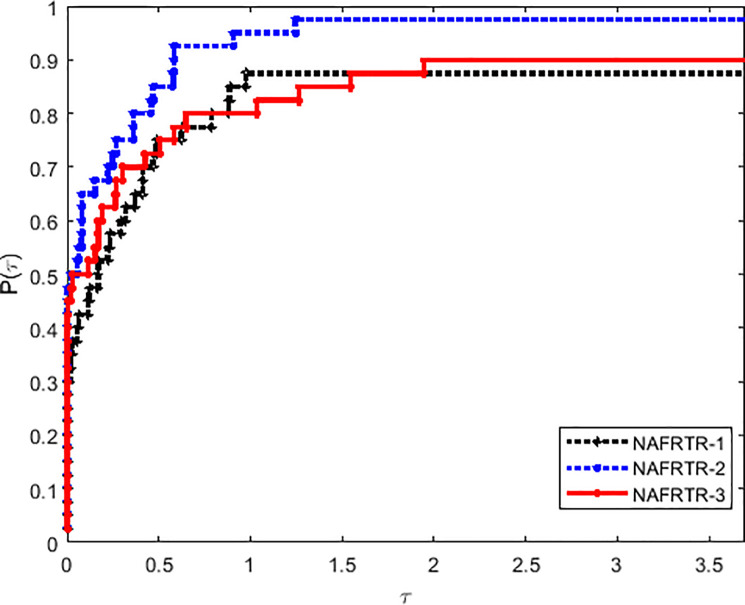
Performance profiles on the basis of the number of gradient evaluations.

In this way, it has to be noted that the selection of initial trust region radius has a great influence in the efficiency of the algorithm. Tables 1 and 2 and Figs 1–3 imply that NAFRTR-2 solves more than 95% of the problems with the minimum number of failures compared with the other two initial radius. At a glance, we choose the initial trust region radius Δ_0_ = 10, as a given parameter for the following algorithm.

**Table 1:** The numerical results of algorithm with various initial radius.

			NAFRTR-1	NAFRTR-2	NAFRTR-3
No.	Problem	*n*	*n*_*f*_/*n*_*i*_		
1	Extended Rosenbrock	200	1191/596	1260/631	-
2	Generalized Rosenbrock	100	1719/860	875/438	1793/897
3	Extended Beale	200	31/16	37/19	35/18
4	Perturbed Quadratic	36	89/45	87/44	89/45
5	Penalty	20	232/117	214/108	289/145
6	Raydan 1	200	511/256	539/270	519/260
7	Raydan 2	200	15/8	13/7	11/6
8	Diagonal 2	500	267/134	393/197	263/132
9	Generalized Tridiagonal 1	500	53/27	53/27	51/26
10	Extended Tridiagonal 1	500	39/20	35/18	25/13
11	Extended TET	500	17/9	15/8	15/8
12	Diadonal 4	500	9/5	11/6	7/4
13	Extended Himmelblau	1000	29/15	29/15	29/15
14	Extended Powell	200	697/349	806/420	806/420
15	Full Hessian 3	1000	10/8	9/8	9/8
16	Extended BD1	1000	174/88	183/92	169/87
17	Perturbed Quadratic Diagonal	500	-	261/131	761/383
18	Quadratic QF1	500	1734/1309	1190/708	1625/1004
19	ARWHEAD	500	-	43/22	31/16
20	NONDIA	1000	59/30	89/45	93/47
21	DQDRTIC	1000	27/14	35/18	31/16
22	EG2	200	-	60/53	219/204
23	Broyden.Tridiation	1000	579/348	384/202	Failed
24	Almost Perturbed Quadratic	192	303/152	717/360	340/171
25	Perturbed Tridiagonal Quadratic	500	551/276	559/280	553/277
26	Extended DENSCHF 35	1000	41/21	35/18	39/20
27	SINCOS 36	1000	-	61/31	-
28	HIMMELH	1000	9/8	8/8	9/8
29	EDENSCH	1000	35/18	25/13	29/16
30	CUBE	1000	3127/1564	Failed	4517/3759
31	BDEXP	1000	53/27	47/24	51/26
32	QUARTC	3000	49/25	47/24	45/23
33	Extended PSC1	3000	43/22	35/18	33/17
34	Quadratic QF2	200	-	686/641	587/498
35	Extended Tridiagonal 2	3000	13/7	9/5	11/6
36	Diagonal 7	3000	18/11	16/10	13/9
37	Diagonal 8	1000	11/8	7/6	14/9
38	Diagonal 9	1000	764/527	571/286	-
39	HIMMELBG	3000	45/23	43/22	43/22
40	Generalized Quartic	3000	185/93	294/148	157/79

**Table 2:** The numerical results of algorithm with various initial radius.

	NAFRTR-1		NAFRTR-2		NAFRTR-3	
No.	CPU	*f*(*x*)	CPU	*f*(*x*)	CPU	*f*(*x*)
1	46.637771	4.583815e-15	36.260390	1.384158e-14	-	-
2	32.602418	3.892592e-14	21.731327	6.119815e-16	44.396016	5.507727e-14
3	0.312776	9.151778e-15	0.417478	5.280240e-16	0.380251	2.942473e-16
4	0.138253	2.782988e-15	0.237082	1.217614e-15	0.131196	1.526180e-15
5	0.316953	1.577771e-04	0.296930	1.577776e-04	0.431847	1.577771e-04
6	14.254856	2.010000e+03	15.063743	2.010000e+03	19.106114	2.010000e+03
7	0.254316	2.000000e+02	0.247048	2.000000e+02	0.136990	2.000000e+02
8	5.763769	1.985454e+01	74.892615	2.603690e+01	5.001542	1.985454e+01
9	5.451341	3.966591e+03	4.935677	3.967692e+03	0.165416	3.817879e+02
10	8.688504	2.466836e+03	4.995965	2.466836e+03	2.409760	2.466836e+03
11	5.865066	6.398167e+02	2.679179	6.398167e+02	1.484941	6.398167e+02
12	3.361856	1.839389e-15	5.634865	4.239477e-16	0.699865	2.122872e-18
13	36.222765	1.689856e-18	13.036732	4.208999e-19	13.334244	1.933005e-14
14	32.856925	9.547051e-10	47.697912	7.151489e-10	45.136583	7.151489e-10
15	15.867802	-2.043783e+02	9.285441	-2.043783e+02	12.674910	-2.043783e+02
16	143.85266	3.173473e-14	169.873944	4.643901e-14	209.655313	4.774523e-14
17	-	-	99.263988	1.907005e-13	451.369860	5.935574e-13
18	2083.100452	-1.000000e-03	813.043727	-1.000000e-03	1601.900050	-1.000000e-03
19	-	-	5.321657	1.221245e-15	3.131264	0.000000e-00
20	63.320012	8.218692e-12	120.302875	7.868990e-12	103.979064	5.132460e-12
21	23.49461	1.123477e-15	25.847274	1.805866e-18	24.039933	1.047419e-16
22	-	-	1.708625	-1.999490e+02	-	-
23	257.32042	2.468230e-03	80.194234	3.617596e-03	-	-
24	22.803333	1.123477e-15	33.488053	4.842693e-15	5.209549	1.985708e-15
25	128.644854	1.265847e-15	120.333364	8.433160e-16	126.147125	1.137918e-15
26	45.951071	1.904287e-17	20.745042	1.082076e-15	54.596513	8.136256e-18
27	-	-	4.489182	2.000000e-00	14.938248	-1.990000e-00
28	16.206452	-5.000000e+00	14.705810	-5.000000e+00	16.402178	-5.000000e+00
29	106.474900	7.885800e+04	63.472192	7.885800e+04	42.172235	7.885800e+04
30	195.604987	1.377615e-07	-	1.644049e-07	235.02607	6.638982e-09
31	72.153478	1.387487e-06	59.224635	8.209430e-07	74.321070	6.621920e-07
32	627.723800	1.154419e-08	481.009080	2.181911e-08	553.410735	1.016167e-08
33	815.975027	1.159799e+03	412.175439	1.159799e+03	364.387727	1.159799e+03
34	-	-	123.673018	-1.000624e+00	79.375143	-1.000624e+00
35	109.413710	1.168798e+03	59.507554	1.168798e+03	50.076078	1.168798e+03
36	278.038612	-8.168486e-01	206.793213	-8.168486e-01	185.161129	-8.168486e-01
37	163.555300	-4.804530e-01	5.985675	-4.804530e-01	108.282310	-4.804530e-01
38	2767.400749	1.347062e-20	763.733536	8.809301e-22	-	-
39	327.576654	1.593518e-08	151.893298	1.720954e-08	301.941347	1.689760e-08
40	3077.534970	2.394372e-14	5754.187327	1.065323e-15	1686.464044	2.508580e-17

**Fig 4:**
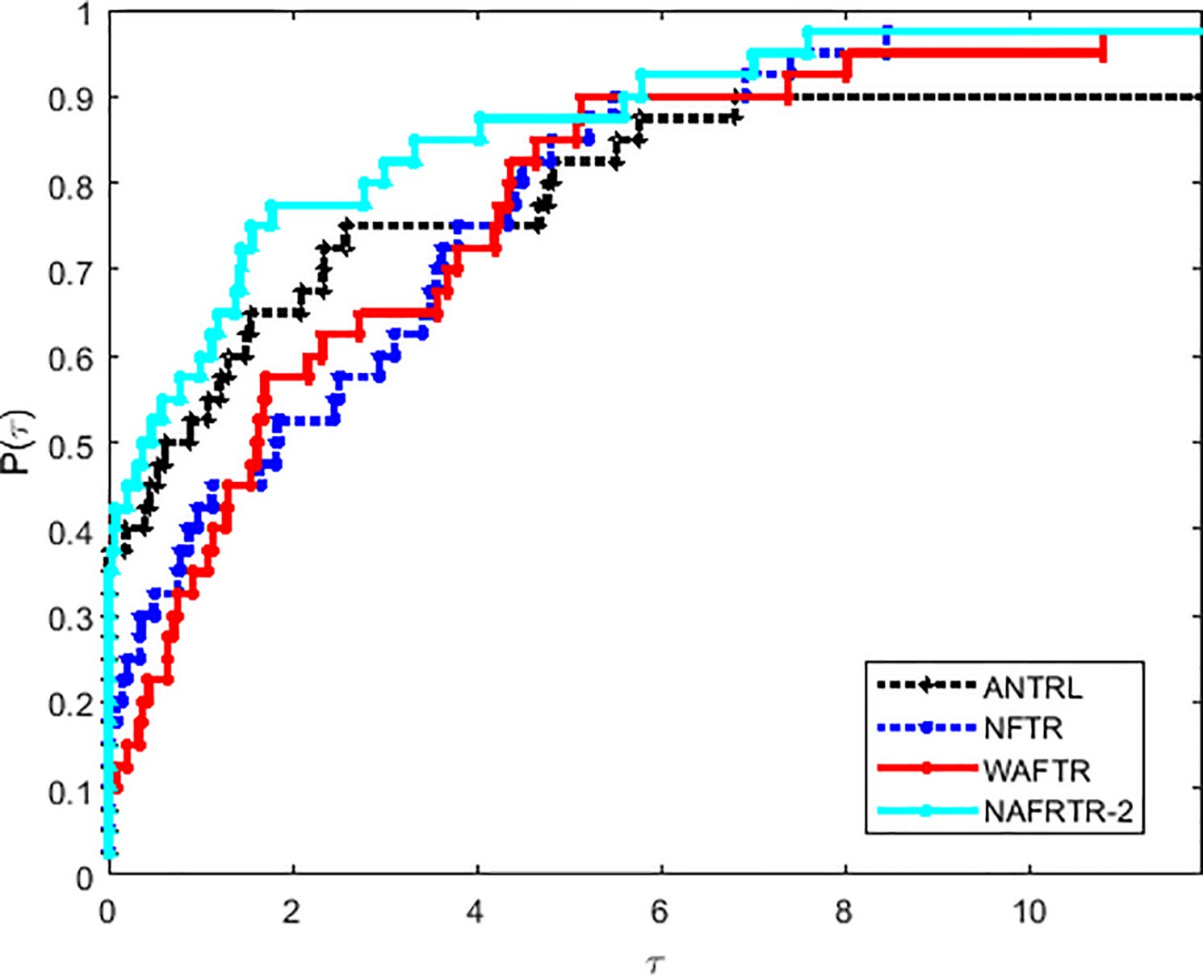
Performance profiles of these algorithms on the basis of running time (in seconds).

**Fig 5:**
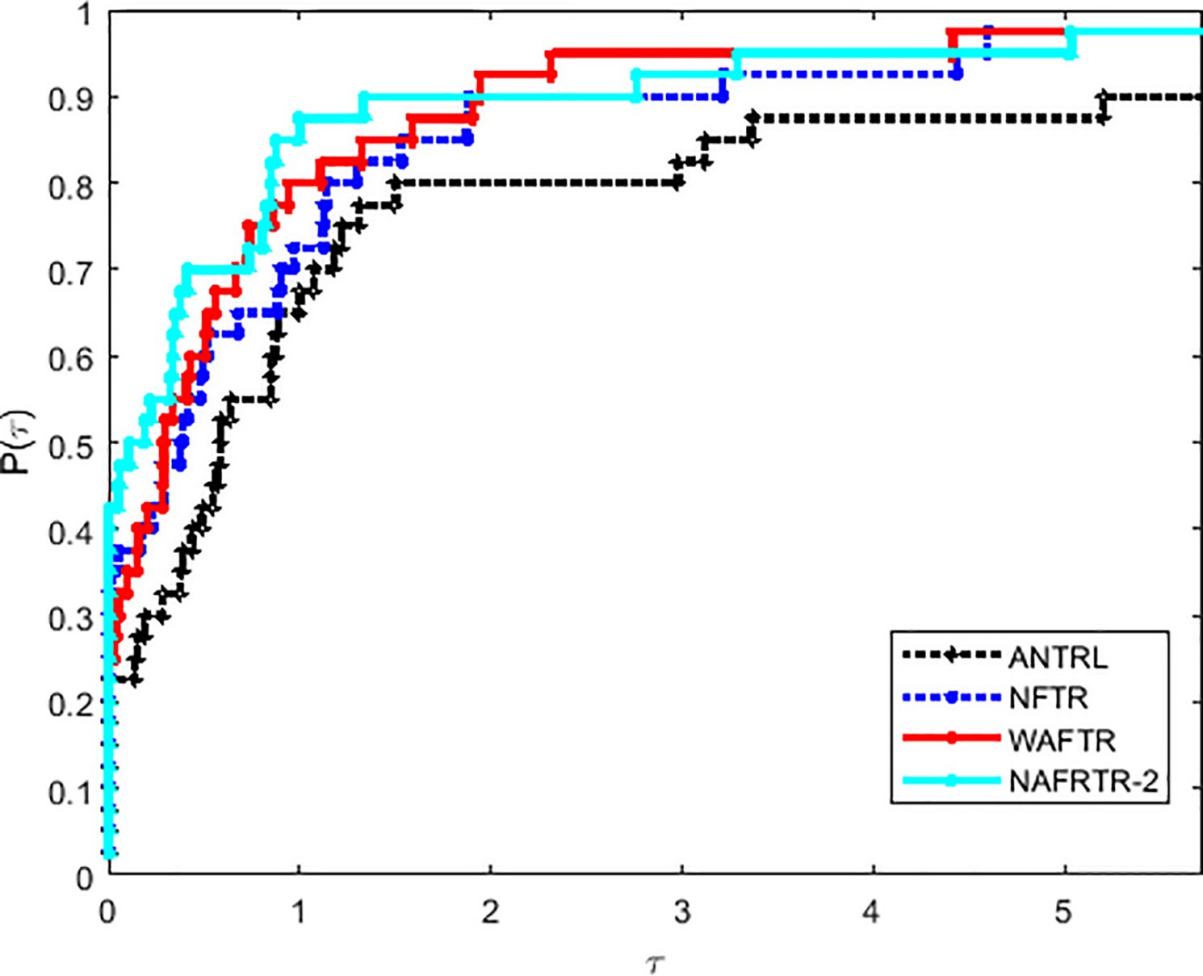
Performance profiles of these algorithms on the basis of the number of function evaluations.

**Fig 6:**
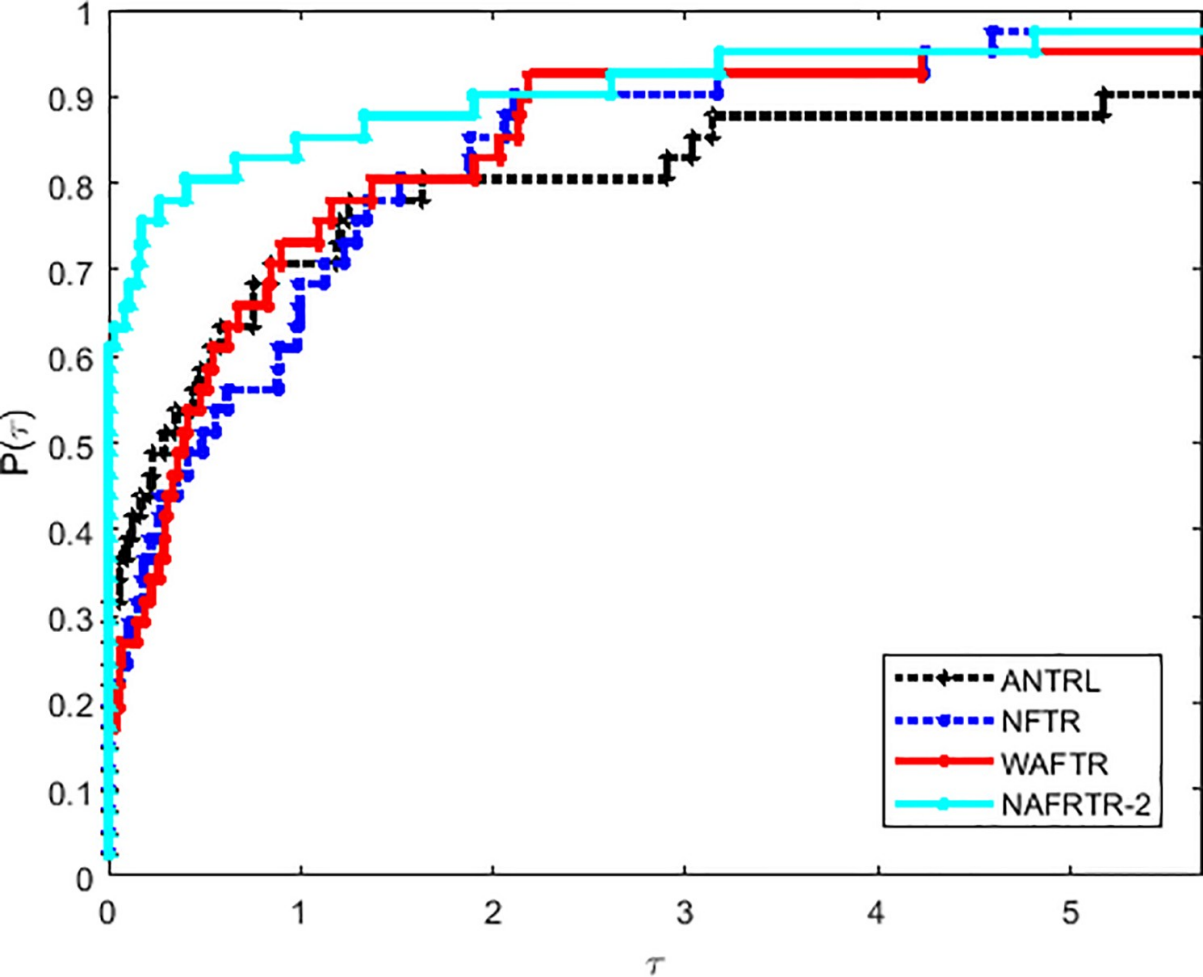
Performance profiles of these algorithms on the basis of the number of gradient evaluations.

Moreover, as shown in Figs 4–6 and Table 2, the NAFRTR-2 is the best solver, in the field of CPU, *n*_*f*_ and *n*_*i*_, about 98% problems respectively. As it is clear that NAFRTR-2 is effective and obtains better performance profiles by comparing with ANTRL, NFTR and WAFTR. Based on the above main observations, the modified trust region method comes out to be fairly effective for unconstrained optimization.

Moreover, as shown in Figs 4–6 and Table 3, the RNATR-A is the best solver, in the field of CPU, *n*_*f*_ and *n*_*i*_, about 98% problems respectively. As it is clear that NAFRTR-2 is effective and obtains better performance profiles by comparing with ANTRL, NFTR and WAFTR. Based on the above main observations, the modified trust region method comes out to be fairly effective for unconstrained optimization.

**Table 3:** Numerical comparisons on a subset of test problems.

No.	*n*	ANTRL33	NFTR	WA FTR57	NAFRTR-2 84
1	200	-	1190/626	129/70	1260/631
2	100	1984/1000	3203/1609	1069/543	875/438
3	200	32/17	29/25	48/44	37/19
4	36	-	116/81	107/78	87/44
5	1000	85/43	2057/1039	121/66	113/57
6	200	1054/578	507/254	501/251	539/270
7	200	478/252	17/9	15/8	13/7
8	500	12/7	260/132	256/131	393/197
9	500	461/238	153/77	55/28	53/27
10	500	51/27	41/24	36/22	35/18
11	500	35/19	15/8	15/8	15/8
12	500	17/9	6/5	10/9	11/6
13	1000	28/16	69/55	70/56	29/15
14	200	-	1138/588	978/540	806/420
15	1000	14/8	9/8	9/8	9/8
16	1000	27/15	27/17	49/28	183/92
17	500	2127/1072	207/140	275/172	261/131
18	500	1356/694	-	2567/1481	1190/708
19	500	46/24	34/25	169/96	43/22
20	1000	67/34	107/86	258/139	89/45
21	1000	40/22	53/44	27/25	35/18
22	1000	51/27	74/63	34/34	34/34
23	1000	539/273	498/310	427/289	384/202
24	192	3389/1706	430/228	634/333	717/360
25	500	621/329	1048/553	704/373	559/280
26	1000	30/17	110/73	112/75	35/18
27	1000	79/43	85/57	68/38	61/31
28	1000	16/9	8/8	8/8	8/8
29	1000	24/13	27/14	46/29	23/12
30	1000	-	3179/1649	5040/2632	3403/1702
31	1000	47/25	26/25	26/25	47/24
32	3000	47/25	48/29	26/24	47/24
33	3000	42/23	29/20	28/18	35/18
34	200	343/172	750/716	1033/782	686/641
35	3000	9/5	11/6	11/6	9/5
36	3000	17/9	14/9	15/10	16/10
37	1000	13/7	10/7	10/7	7/6
38	1000	598/368	327/254	439/261	571/286
39	3000	107/68	95/48	44/23	43/22
40	3000	386/249	359/295	-	294/148

## 5. Conclusions

In this paper, making proper use of the filter technique, a new trust region method has been proposed that the trust region radius takes into account the gradient information of the function and the solution of the trust region subproblem. To some extent, it is more reasonable to adopt the convex combination of nonmonotone trust region ratio and retrospective ratio. In addition, the approximation of the Hessian matrix is updated by an improved quasi-Newton formula. From the theoretical view, the new algorithm keeps global convergent and superlinear convergent. Numerical experiments show the availability of the new proposed algorithm in accordance with the Dolan-Moré performance profile.
